# Construction and Validation of a Long‐Term Visual Prognosis Prediction Model for Proliferative Diabetic Retinopathy After Vitrectomy Based on Machine Learning

**DOI:** 10.1002/edm2.70314

**Published:** 2026-09-07

**Authors:** Xiaolu Wang, Jinghua Shi, Yan Gao, Tiantian Li, Xuehong Han, Lixia Guo, Tingting Jia, Yan Wang

**Affiliations:** ^1^ School of Nursing Shanxi Medical University People's Republic of China; ^2^ Department of Nursing Shanxi Eye Hospital People's Republic of China; ^3^ Department of Vitreoretinopathy Shanxi Eye Hospital People's Republic of China

**Keywords:** diabetic retinopathy, predictive model, vitrectomy

## Abstract

**Introduction:**

This study aims to develop a machine‐learning‐based risk prediction model for a long‐term visual prognosis in patients with proliferative diabetic retinopathy (PDR) following pars plana vitrectomy (PPV).

**Methods:**

We analysed 609 PDR patients (609 eyes) who underwent PPV at Shanxi Eye Hospital from January 1, 2022, to January 1, 2025. The dataset was randomly split into training and validation sets at an 8:2 ratio. Candidate risk factors were identified using LASSO regression, followed by multivariate logistic regression to determine independent predictors. Machine learning algorithms were then trained to construct the prediction model, with performance evaluated by AUC‐ROC, accuracy, precision, recall, and F1 score. Calibration was assessed via calibration curves, and clinical utility by decision curve analysis (DCA).

**Results:**

LASSO regression and multivariate logistic regression indicated that several factors influenced postoperative long‐term visual prognosis. These included Renal Insufficiency (OR = 6.932, 95% CI: 3.394–14.158), Preoperative iris neovascularization (OR = 7.674, 95% CI: 3.699–15.920), Silicone oil tamponade (OR = 2.799, 95% CI: 1.641–4.707), Indirect bilirubin (IBIL) (OR = 0.902, 95% CI: 0.829–0.981) (*p* < 0.05). LightGBM was found to be the best for predicting postoperative long‐term visual prognosis risk in PDR patients. The LightGBM model demonstrated good calibration in both training and validation sets. DCA of the validation set showed clinical net benefit at low‐to‐moderate risk thresholds, outperforming both ‘treat‐all’ and ‘treat‐none’ strategies.

**Conclusions:**

In conclusion, this study developed a machine learning‐based prognostic model for long‐term visual prognosis in PDR patients after PPV, visualized as a proof‐of‐concept web calculator to assist clinical staff in early risk identification and support personalised treatment planning, pending external validation and prospective evaluation.

## Graphical abstract

This study developed and validated a machine learning‐based prognostic model for predicting long‐term visual outcomes after pars plana vitrectomy (PPV) in patients with proliferative diabetic retinopathy.

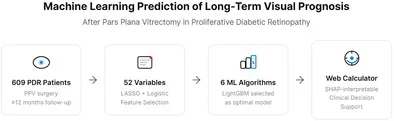

## 1 Introduction

Diabetic retinopathy (DR) is a common microvascular complication of diabetes and a leading preventable cause of blindness in working‐age adults aged 20 to 74 years [1]. In Asia, the prevalence of DR among Type 2 Diabetes patients is 25%, with PDR accounting for 15% [2, 3]. Worldwide, the prevalence rates of DR and PDR are 35.4% and 7.5%, respectively [4]. The early stage of DR is often hidden and has no symptoms. However, the disease can quickly progress to PDR, often without the patient's notice. This may cause serious complications such as neovascular glaucoma, vitreous haemorrhage, and tractional retinal detachment, leading to irreversible vision impairment or blindness [5].

At present, PPV is the primary treatment method for patients with PDR. By eliminating neovascularization, resolving vitreous haemorrhage, and reducing retinal traction, it can effectively restore patients' vision and reduce vision loss from PDR progression. Thereby maintaining the visual function and quality of life of patients [6]. However, due to many factors such as the relatively severe condition of PDR patients, numerous underlying diseases, long treatment cycles of fundus laser or anti‐VEGF, and poor treatment compliance of patients, the current status and prognosis of postoperative visual function of patients are complex and changeable [7, 8]. The goal of PDR treatment should not be limited solely to preventing blindness; it should also focus on improving and maintaining the patient's long‐term good vision after vitrectomy to ensure the effectiveness of surgical treatment. Therefore, exploring risk factors for the long‐term visual prognosis of PDR patients undergoing PPV and implementing targeted interventions on these risk factors in postoperative patients at an early stage are of great significance for reducing the blindness rate and medical burden caused by PDR, conserving medical resources, and improving the overall health of society.

However, it is difficult to fully capture the overall characteristics of these patients' postoperative visual prognosis. The scope of clinical data collection is relatively limited, and the completeness and representativeness of the data are poor [9, 10, 11]. To address the aforementioned limitations, this study determined an appropriate sample size using a standardized calculation formula and conducted a comprehensive screening of electronic medical record data. Postoperative patients were followed up for over 12 months to continuously monitor their disease progression. Through a holistic evaluation of relevant influencing factors before and during surgery, this study identified key factors affecting long‐term visual outcomes following vitrectomy for PDR.

In recent years, machine learning algorithms have been widely applied in the medical field, demonstrating significant advantages in clinical data processing, analysis, and visualisation [12]. Based on these findings, we developed and validated a prediction model for long‐term visual prognosis in PDR patients after diabetic vitrectomy, employing multiple machine learning algorithms. The model aims to predict postoperative visual outcomes and support the development of personalised treatment plans by targeting identifiable risk factors. Additionally, to further standardise the management and follow‐up of ophthalmic patients and ensure timely delivery of personalised interventions and health education, we performed a visual interpretation of the optimal model using SHAP (SHapley Additive exPlanations) diagrams and a web‐based calculator.

## 2 Method

### 2.1 Study Population

A total of 609 patients (609 eyes) with proliferative diabetic retinopathy who underwent first‐time vitrectomy at Shanxi Eye Hospital from January 1, 2022, to January 1, 2025, were included in this study. The inclusion criteria are as follows: (1) The ophthalmic examination shows that the PDR diagnostic criteria in the clinical diagnosis and treatment guidelines for diabetic retinopathy are met; (2) Undergo the first PPV treatment due to PDR and be at least 18 years old; (3) Follow‐up for no less than 12 months; (4) Only one eye from each patient was included in the study. If both eyes met the inclusion criteria, the eye with more severe DR was selected for inclusion according to the following rules: priority was given to the eye with more severe DR. If the severity of DR was identical, the eye with poorer visual acuity was included. (5) The case data information during the follow‐up period was complete. Patients with glaucoma, age‐related macular degeneration, and other retinal vascular diseases affecting fundus examination, as well as those with a previous history of ocular surgeries other than cataracts, were excluded from the study.

### 2.2 The Definition of Outcome Metrics

Patients were screened according to the inclusion and exclusion criteria to construct the research cohort. Best‐corrected visual acuity (BCVA), which assesses the sharpest vision a patient can achieve with correction, was measured using the Snellen visual acuity chart and analysed on the logMAR (logarithm of the minimum angle of resolution) scale. This study aims to accurately assess the long‐term visual prognosis of patients after surgery. We designate the last follow‐up time point as the pivotal time point for evaluating visual prognosis. The follow‐up period must be no less than 12 months (here, ‘long‐term vision’ is defined as the visual acuity measured at 12 months post‐surgery. Previous studies have demonstrated that patients can attain stable vision after this period [9]) to ensure there is ample time to observe changes in patients' vision. In this study, logarithmic minimum angular resolutions of 1.9, 2.3, 2.7 and 3.0 were assigned to digital, manual, photosensitive and non‐photosensitive categories, respectively, in order to describe different visual conditions more clearly. Based on the aforementioned assignment criteria for different visual acuity conditions, the BCVA at the last follow‐up was compared to the preoperative BCVA. The visual prognosis was determined according to the LogMAR change and categorized into a good prognosis group and a poor prognosis group using the dichotomy method (where 0 denotes good and 1 denotes poor).

(1) Good prognosis group: If the LogMAR value decreases by 0.3 units or more, indicating an improvement in visual acuity compared to the preoperative level, the patient is classified into the good prognosis group.

(2) Poor prognosis group: A patient can be classified into the poor prognosis group when either of the following two conditions is met: Firstly, if the LogMAR value increases by more than 0.3 units, indicating a deterioration in vision compared to the preoperative state; secondly, if the decrease in the LogMAR value is less than 0.3 units, or the increase is less than 0.3 units, both of which suggest that vision has not improved compared to the preoperative level.

### 2.3 Sample Size Estimation

Based on the sample size calculation formula in the R pmsampsize package, this study expects to include no more than 15 variables after Logistic regression screening for model construction and to select the prediction model sample size calculation type (type = ‘b’) for binary classification outcomes. The number of preset prediction model parameters (*n* = 15) and the C‐index of previous studies on prognosis risk prediction models for PDR patients after PPV were set at 0.84 [13]. That is, if pmsampsize (type = ‘b’, cstatistic = 0.84, parameters = 15, prevalence = 0.303) is set, the sample size of this study should be at least 385 research subjects and include 117 outcome events. Considering a 10% sample failure rate, the minimum required sample size is 385 ÷ (1–0.1) = 428 cases. The sample size for this study is reasonable.

### 2.4 Assessment of Covariates

The selection of variables is based on comprehensive literature review results and clinical relevance. Data collection is conducted through the hospital's electronic medical record system. General information includes age, systolic blood pressure, diastolic blood pressure, body mass index (BMI), duration of diabetes, age at diabetes diagnosis, gender, marital status, and place of residence, among others. Surgery‐related factors are divided into two parts: preoperative and intraoperative. Preoperative factors encompass the operated eye, the stage of diabetic retinopathy (DR Stage), the preoperative visual acuity of the contralateral eye (LogMAR), the preoperative intraocular pressure of the operated eye (mmHg), and the presence or absence of iris neovascularization prior to the operation, among others. Intraoperative factors include whether cataract surgery is performed concurrently during the operation and the type of intraocular filler utilized during the procedure, among other considerations. Biochemical indicators include glycated haemoglobin, monocytes, lymphocytes, platelets, haematocrit, fasting blood glucose, uric acid, creatinine, and so forth. Based on serum creatinine results, the glomerular filtration rate (eGFR) was calculated using the CKD Epidemiology Collaboration equation [14].

### 2.5 Statistical Analysis

The data were statistically analysed using IBM SPSS 27.0 and R 4.4.3 software. For missing data, multiple imputation was performed using the ‘mice’ package in R. The dataset was split into an 8:2 ratio using the random sampling method. Data from 487 patients (487 eyes) served as the training set for constructing and validating the model, while data from 122 patients (122 eyes) were utilized as the validation set for the model's internal validation.

#### 2.5.1 Variable Selection

Measurement data using mean ± standard deviation ($\bar{x}\pm s$) or the median and quartiles *M* (*P*25, *P*75), and the count data described in frequency, percentage (%). Continuous variables were tested for normality using the *Kolmogorov–Smirnov* test (K‐S test). Data with normal distributions were compared between groups using the independent‐samples *t*‐test, and data with non‐normal distributions were tested using the rank‐sum test. The count data were analysed using the *χ* ^2^ test. Univariate Logistic regression analysis was conducted on the training set data. Incorporate relevant variables into the LASSO regression model to conduct a preliminary screening of predictive factors. The ‘glmnet’ package in R was used for minimum absolute value convergence and LASSO regression to determine the optimal LASSO parameter *λ*. Screen for screen characteristic variables to reduce multicollinearity. The above variables were incorporated into the binary Logistic regression model. The risk factors with statistical significance (*p* < 0.05) in the multivariate regression analysis were used as predictors, and whether the prognosis was good was taken as the target variable to construct a machine learning prediction model.

#### 2.5.2 Model Training and Validation

The purpose of this study is to predict the prognostic risk of PDR patients after PPV treatment. The outcome grouping is a binary classification into the good‐prognosis and poor‐prognosis groups, which falls within the application scope of supervised learning algorithms. Therefore, Decision Tree (DT), Random Forest (RF), Support Vector Machine (SVM), Multilayer Perceptron (MLP), Logistic Regression (LR), and Light Gradient Boosting Machine (LightGBM) were used to establish a prognostic risk prediction model for patients with PDR after PPV. The training set is used to find the optimal parameters via grid search and 10‐fold cross‐validation to construct the model. Among the evaluation metrics for binary classification models, the confusion matrix is the most intuitive and comprehensive measure of model performance. It can visually present the degree of confusion between the predicted and actual categories in the dataset in tabular form and is used to draw the Receiver Operating Characteristic Curve, the foundation of ROC analysis. The performance of the validation set model is mainly evaluated by the area under the ROC curve (AUC). In addition, this study assessed the accuracy and sensitivity of model predictions using accuracy rate, precision rate, recall rate, and F1 score, and compared each model to select the optimal one. The calibration performance of the optimal model's validation set was assessed using calibration curves. Additionally, decision curve analysis (DCA) was performed to evaluate the clinical utility of the LightGBM model by quantifying the net benefit across a range of risk thresholds.

#### 2.5.3 Model Interpretation and Visualisation

Visualize the results of the optimal model using SHAP (Shapley Additive exPlanations) graphs. As a model‐independent local interpretation framework, SHAP can quantify each feature's contribution to the model's prediction for a given sample by calculating each feature's SHAP value and visually presenting the relationship between the feature and the prediction in a web calculator [15].

## 3 Result

### 3.1 Preprocessing Result

**FIGURE 1:**
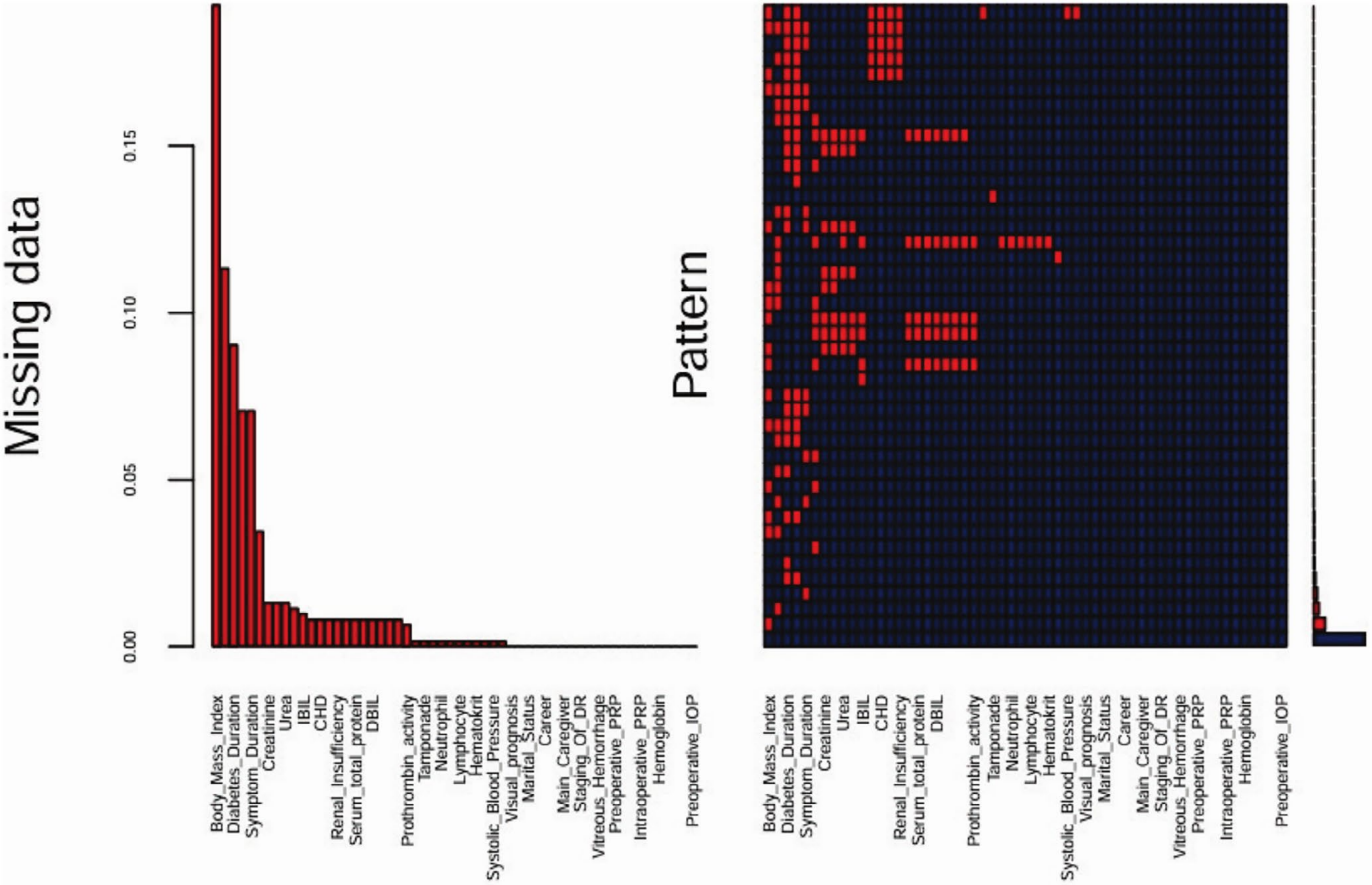
The missing data of covariates.

This study included 52 covariates (see Table S1). As shown in Figure 1, the missing variables are presented. The bar chart illustrates the proportions of data loss across different covariates; the *Y*‐axis shows the proportion of loss for each covariate. The Body Mass Index (BMI) variable has the highest proportion of missing values (19.2%). The correct figure shows missing data in red and no missing data in blue. For variables with missing values, multiple imputation is used to estimate them.

### 3.2 The Baseline Characteristics of the Research Subjects

To avoid differences between the training and validation sets caused by random splitting, descriptive statistics were conducted on the variables in both sets to compare the distributions of the variables between the two groups. The research results indicate that there are no differences in the measured variables between the two groups. Therefore, random grouping is a reasonable approach. As shown in Tables 1, 2, 3.

**TABLE 1:** Baseline characteristics of general data in the training set and validation set.

Variable	Training cohort (*n* = 487)	Validation cohort (*n* = 122)	*p*
Age (years)	57.00 (50.00, 65.00)	58.00 (50.00, 64.00)	0.936
Systolic blood pressure (mmHg)	136.00 (125.00, 140.00)	135.00 (126.50, 140.00)	0.947
Diastolic blood pressure (mmHg)	80.00 (73.00, 86.00)	80.00 (72.00, 86.25)	0.856
Body mass index (kg/m^2^)	24.68 (22.35, 26.96)	24.69 (22.52, 27.77)	0.433
Diabetes duration (years)	12.00 (7.00, 20.00)	12.00 (5.75, 20.00)	0.935
Age at diabetes diagnosis (years)	43.00 (36.00, 51.00)	43.50 (37.00, 50.00)	0.984
Sex			0.556
Female	233.00 (47.80%)	62.00 (50.80%)	
Male	254.00 (52.20%)	60.00 (49.20%)	
Marital status			0.997
Married	467.00 (95.90%)	117.00 (95.90%)	
Unmarried/divorced/widowed	20.00 (4.10%)	5.00 (4.10%)	
Residence			0.763
Country	240.00 (49.30%)	56.00 (45.90%)	
Town	49.00 (10.10%)	12.00 (9.80%)	
City	198.00 (40.70%)	54.00 (44.30%)	
Career			0.286
Peasant	214.00 (43.90%)	44.00 (36.10%)	
Staff	77.00 (15.80%)	20.00 (16.40%)	
Freelance work	31.00 (6.40%)	13.00 (10.70%)	
Retirement	98.00 (20.10%)	30.00 (24.60%)	
Unemployed	67.00 (13.80%)	15.00 (12.30%)	
Insurance type			0.384
Worker's insurance	201.00 (41.30%)	61.00 (50.00%)	
Resident insurance	185.00 (38.00%)	40.00 (32.80%)	
New rural co‐operative medical system	96.00 (19.70%)	20.00 (16.40%)	
Self paying	5.00 (1.00%)	1.00 (0.80%)	
Main caregiver			0.324
Spouse	238.00 (48.90%)	66.00 (54.10%)	
Offspring	231.00 (47.40%)	55.00 (45.10%)	
Solitude	8.00 (1.60%)	0.00 (0.00%)	
Else	10.00 (2.10%)	1.00 (0.80%)	
Diabetes type			0.500
I type	8.00 (1.60%)	1.00 (0.80%)	
II type	479.00 (98.40%)	121.00 (99.20%)	
Diabetes treatment			0.675
Take oral medication alone	135.00 (27.70%)	35.00 (28.70%)	
Insulin alone	93.00 (19.10%)	27.00 (22.10%)	
Oral medication combined with insulin	259.00 (53.20%)	60.00 (49.20%)	
Hypertension			0.314
No	203.00 (41.70%)	57.00 (46.70%)	
Yes	284.00 (58.30%)	65.00 (53.30%)	
Cerebral infarction			0.119
No	452.00 (92.80%)	108.00 (88.50%)	
Yes	35.00 (7.20%)	14.00 (11.50%)	
CHD			0.634
No	442.00 (90.80%)	109.00 (89.30%)	
Yes	45.00 (9.20%)	13.00 (10.70%)	
Renal insufficiency			0.501
No	441.00 (90.60%)	108.00 (88.50%)	
Yes	46.00 (9.40%)	14.00 (11.50%)	

*Note:* Quantitative variables are expressed as mean ± SD or median with quartiles, and qualitative variables are expressed as *n* (%). *p*‐values resulted from independent *t*‐tests for quantitative and Chi‐squared for qualitative variables between the two groups.

**TABLE 2:** Distribution characteristics of surgery‐related factors in the training set and validation set.

Variable	Training cohort (*n* = 487)	Validation cohort (*n* = 122)	*p*
Eye			0.384
Left eye	253.00 (52.00%)	58.00 (47.50%)	
Right eye	234.00 (48.00%)	64.00 (52.50%)	
Staging Of DR			0.087
IV stages	76.00 (15.60%)	11.00 (9.00%)	
V stages	335.00 (68.80%)	85.00 (69.70%)	
VI stages	76.00 (15.60%)	26.00 (21.30%)	
Preoperative contralateral BCVA (LogMAR)	0.60 (0.30,1.00)	0.60 (0.30,1.10)	0.720
Preoperative IOP (mmHg)	15.00 (13.00,17.00)	16.00 (13.00,17.00)	0.725
Preoperative Iris neovascularization			0.091
No	444.00 (91.20%)	105.00 (86.10%)	
Yes	43.00 (8.80%)	17.00 (13.90%)	
Preoperative vitreous hemorrhage			0.478
No	2.00 (0.40%)	0.00 (0.00%)	
Yes	485.00 (99.60%)	122.00 (100.00%)	
Preoperative tractional retinal detachment			0.159
No	437.00 (89.70%)	104.00 (85.20%)	
Yes	50.00 (10.30%)	18.00 (14.80%)	
Preoperative PRP			0.989
No	331.00 (68.00%)	83.00 (68.00%)	
Yes	156.00 (32.00%)	39.00 (32.00%)	
Symptom duration (month)	2.00 (1.00,3.00)	2.00 (1.00,4.00)	0.585
Combined cataract extraction			0.871
No	148.00 (30.40%)	38.00 (31.10%)	
Yes	339.00 (69.60%)	84.00 (68.90%)	
Intraoperative PRP			0.315
No	4.00 (0.80%)	0.00 (0.00%)	
Yes	483.00 (99.20%)	122.00 (100.00%)	
Intravitreal injection			0.592
No	7.00 (1.40%)	1.00 (0.80%)	
Yes	480.00 (98.60%)	121.00 (99.20%)	
Tamponade			0.957
No	313.00 (64.30%)	79.00 (64.80%)	
Silicone oil	151.00 (31.00%)	38.00 (31.10%)	
Gases	23.00 (4.70%)	5.00 (4.10%)	

*Note:* Variables are expressed as *n* (%). *p* values resulted from independent *t*‐tests for quantitative and Chi‐square for qualitative variables between the two groups.

**TABLE 3:** Distribution characteristics of biochemical indicators in the training set and validation set.

Variable	Training cohort (*n* = 487)	Validation cohort (*n* = 122)	*p*
HbA1c (%)	6.60 (5.70, 7.70)	6.50 (5.90, 7.60)	0.936
Monocyte (×10^9^/L)	0.44 (0.36, 0.54)	0.44 (0.37, 0.51)	0.488
Lymphocyte (×10^9^/L)	2.02 (1.62, 2.48)	1.99 (1.67, 2.43)	0.911
Platelet (×10^9^/L)	217.00 (181.00, 264.00)	212.00 (178.75, 258.75)	0.449
Haematocrit (g/L)	40.30 (37.10, 43.70)	40.80 (36.33, 43.83)	0.868
Haemoglobin (g/L)	133.00 (122.00, 146.00)	133.00 (118.00, 145.00)	0.635
Fasting blood glucose (mmol/L)	6.43 (5.08, 8.20)	6.28 (5.11, 8.05)	0.586
Uric acid (μmol/L)	342.00 (286.00, 415.00)	340.00 (281.25, 415.25)	0.711
Creatinine (μmol/L)	70.00 (56.80, 90.00)	68.30 (55.95, 90.43)	0.786
eGFR (mL/min/1.73 m^2^)	92.34 (74.92, 103.84)	93.52 (71.50, 103.61)	0.958
Urea	6.48 (5.19, 8.25)	6.56 (5.19, 8.41)	0.840
Serum albumin (g/L)	40.00 (37.00, 43.00)	40.00 (36.00, 43.00)	0.555
Serum total protein (g/L)	66.00 (62.00, 70.00)	65.00 (61.00, 70.00)	0.307
Sap (U/L)	64.00 (53.00, 80.00)	70.00 (53.00, 84.00)	0.177
IBIL (μmol/L)	5.81 (4.09, 8.12)	5.53 (3.40, 8.57)	0.672
DBIL (μmol/L)	4.62 (3.47, 5.85)	4.71 (3.58, 6.08)	0.611
TBIL (μmol/L)	10.44 (7.85, 13.34)	10.39 (6.98, 14.14)	0.550
Serum alanine aminotransferase (U/L)	16.00 (12.00, 23.00)	16.00 (12.75, 25.25)	0.525
Serum aspartate aminotransferase (U/L)	17.00 (14.00, 20.00)	17.00 (14.00, 21.00)	0.568
Serum b glutamyl transferase (U/L)	17.00 (12.00, 24.00)	14.50 (12.00, 23.00)	0.179
Prothrombin activity	0.98 (0.95, 0.99)	0.97 (0.95, 0.99)	0.362

*Note:* Quantitative variables are expressed as mean ± SD or median with quartiles, and qualitative variables are expressed as *n* (%). *p* values resulted from independent *t*‐tests for quantitative and Chi‐square for qualitative variables between the two groups.

Abbreviations: DBIL, direct bilirubin; eGFR, estimated glomerular filtration rate; HbA1c, glycosylated haemoglobin; IBIL, indirect bilirubin; Sap, serum alkaline phosphatase; TBIL, total bilirubin.

### 3.3 LASSO Regression Is Used for Variable Screening

**FIGURE 2:**
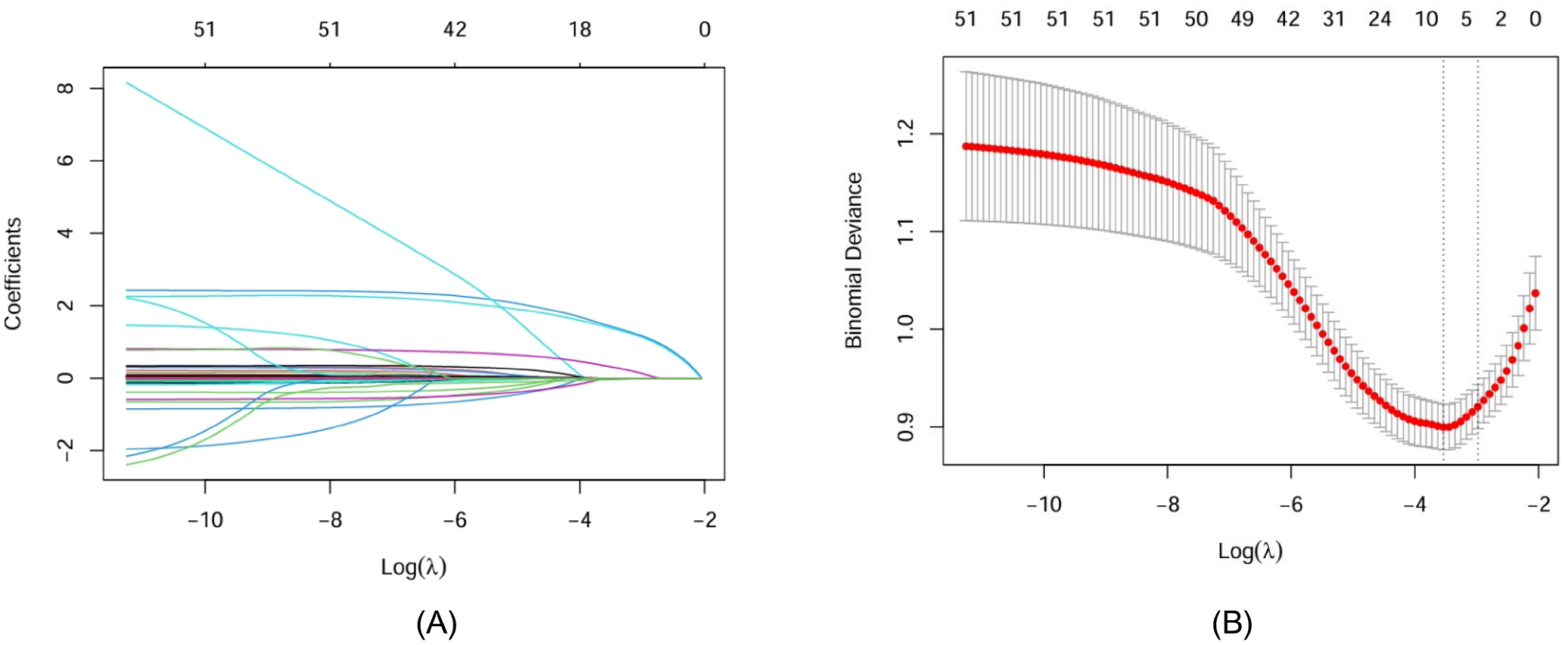
LASSO regression for variable screening.

Given the substantial number of clinical indicators in PDR patients following PPV and the potential for multicollinearity, the poor prognosis status of the training cohort was defined as the dependent variable, while all other variables were treated as independent variables for LASSO regression analysis. The above variables were included for LASSO regression analysis. LASSO compresses and controls the regression coefficients of independent variables by constructing a penalty function. As the penalty coefficient *λ* changes, the predictor variables included in the model gradually decrease, as shown in Figure 2A. As shown in Figure 2B, the error rate of the obtained model is the smallest when the penalty parameter *λ* converges to 0.02910. At this point, the candidate predictive variables screened by LASSO regression were whether there was age (years), renal insufficiency, iris neovascularization, tamponade, Sap (U/L), IBIL (μmol/L), and Serum b glutamyl transferase (U/L).

### 3.4 Binary Logistic Regression Analysis

Eight risk factors were selected via LASSO regression as independent variables for binary Logistic regression analysis. Substitute the continuous variable directly into the original data values; assignment processing is required for binary and unordered categorical variables. The results of binary Logistic regression showed that among the eight screened risk factors, the results indicated that renal insufficiency (1) (OR = 6.932, 95% CI: 3.394–14.158), preoperative iris neovascularization (1) (OR = 7.674, 95% CI: 3.699–15.920), tamponade (1) (OR = 2.799, 95% CI: 1.641–4.707), IBIL (OR = 0.902, 95% CI: 0.829–0.981) were statistically significant (*p* < 0.05), as detailed in Table 4.

**TABLE 4:** Results of multivariate logistic regression analysis on poor prognosis of DR patients after PPV (*n* = 487).

Variable	*β*	SE	Wald *χ* ^2^	*p*	OR	95% CI	
Age	0.029	0.013	5.042	0.025	1.029	1.004	1.055
Renal insufficiency (1)	1.936	0.364	28.232	< 0.001	6.932	3.394	14.158
Preoperative iris neovascularization (1)	2.038	0.372	29.951	0.001	7.674	3.699	15.920
Tamponade (1)	1.022	0.269	14.448	< 0.001	2.779	1.641	4.707
Sap (U/L)	0.004	0.006	0.531	0.466	1.004	0.993	1.015
IBIL (μmol/L)	−0.103	0.043	5.738	0.017	0.902	0.829	0.981
Serum b glutamyl transferase (U/L)	0.009	0.007	1.936	0.164	1.009	0.996	1.022

*Note:* *p* < 0.05.

### 3.5 Model Training and Validation

In this study, 10‐fold cross‐validation was used on the training set, and models were constructed using six algorithms. Eventually, six prediction models for the prognosis risk of PDR patients after PPV were obtained. To ensure model stability, the average 10‐fold cross‐validation score for each algorithm was used as the final result for accuracy, precision, recall rate, and F1 score. The differences were all statistically significant (*p* < 0.001), and the 10‐fold cross‐validation evaluation results of each algorithm are shown explicitly in Table 5.

**TABLE 5:** Evaluation of the 10‐fold cross‐validation results of the machine learning algorithm training set.

Model	Accuracy		Precision		Recall		F1 score		Average AUC	
	$\bar{x}$ ± s	95% CI	$\bar{x}$ ± s	95% CI	$\bar{x}$ ± s	95% CI	$\bar{x}$ ± s	95% CI	$\bar{x}$ ± s	95% CI
DT	0.746 ± 0.049	0.711–0.781	0.730 ± 0.057	0.689–0.771	0.878 ± 0.050	0.842–0.913	0.794 ± 0.027	0.775–0.813	0.741 ± 0.064	0.696–0.787
RF	0.795 ± 0.036	0.770–0.820	0.832 ± 0.037	0.806–0.858	0.928 ± 0.047	0.894–0.962	0.876 ± 0.024	0.859–0.893	0.750 ± 0.131	0.656–0.844
SVM	0.766 ± 0.046	0.734–0.799	0.748 ± 0.077	0.692–0.803	0.886 ± 0.066	0.838–0.933	0.807 ± 0.044	0.775–0.838	0.804 ± 0.052	0.766–0.841
MLP	0.769 ± 0.056	0.729–0.810	0.767 ± 0.074	0.715–0.820	0.844 ± 0.041	0.814–0.873	0.802 ± 0.051	0.766–0.838	0.839 ± 0.050	0.803–0.875
LR	0.797 ± 0.042	0.767–0.827	0.821 ± 0.037	0.794–0.848	0.948 ± 0.036	0.922–0.974	0.879 ± 0.028	0.860–0.899	0.771 ± 0.11	0.692–0.849
LightGBM	0.803 ± 0.047	0.770–0.836	0.811 ± 0.050	0.775–0.846	0.979 ± 0.026	0.961–0.998	0.886 ± 0.030	0.865–0.907	0.763 ± 0.125	0.674–0.853

**FIGURE 3:**
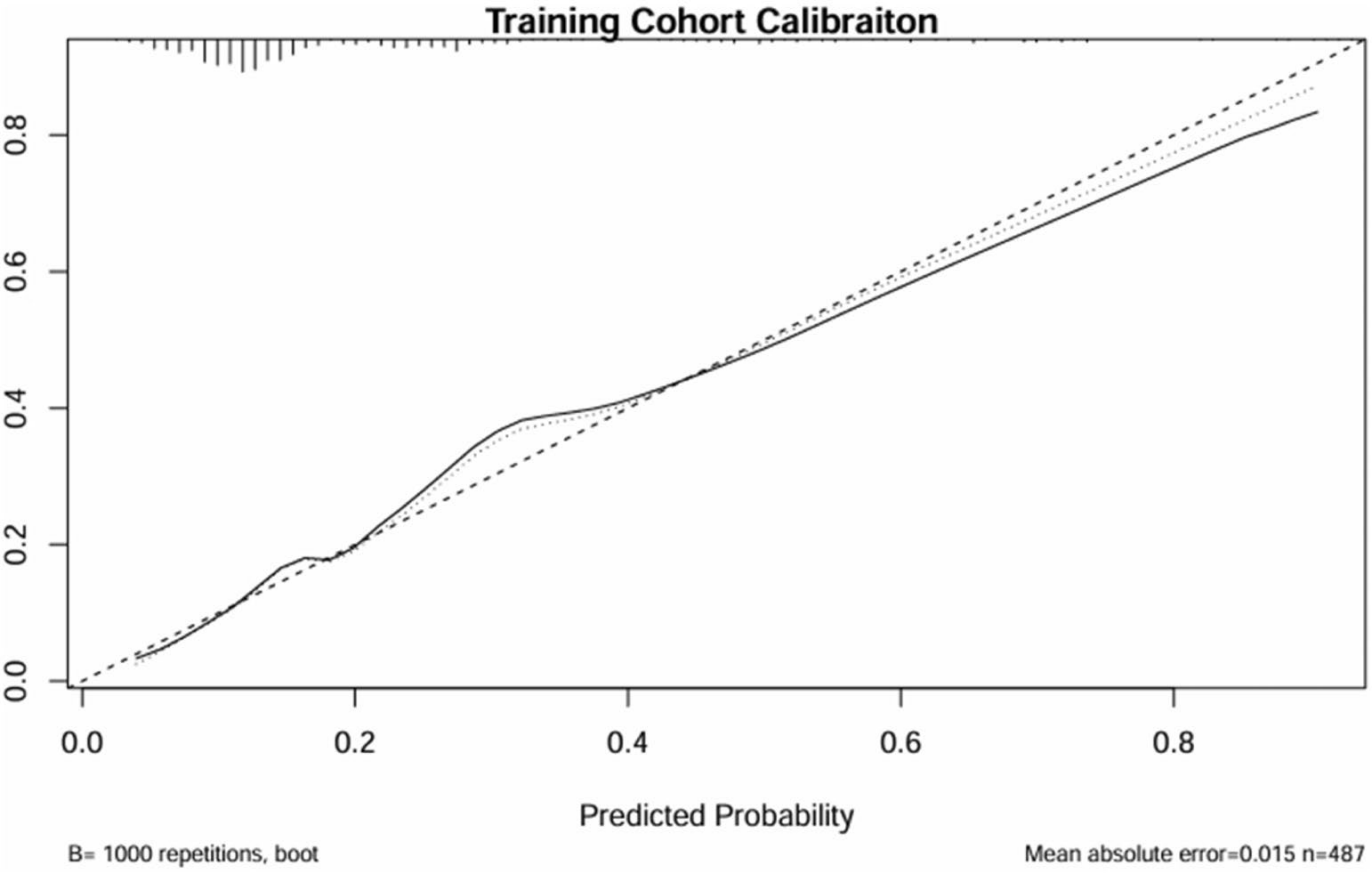
Calibration curve of the LightgBM model training set.

**FIGURE 4:**
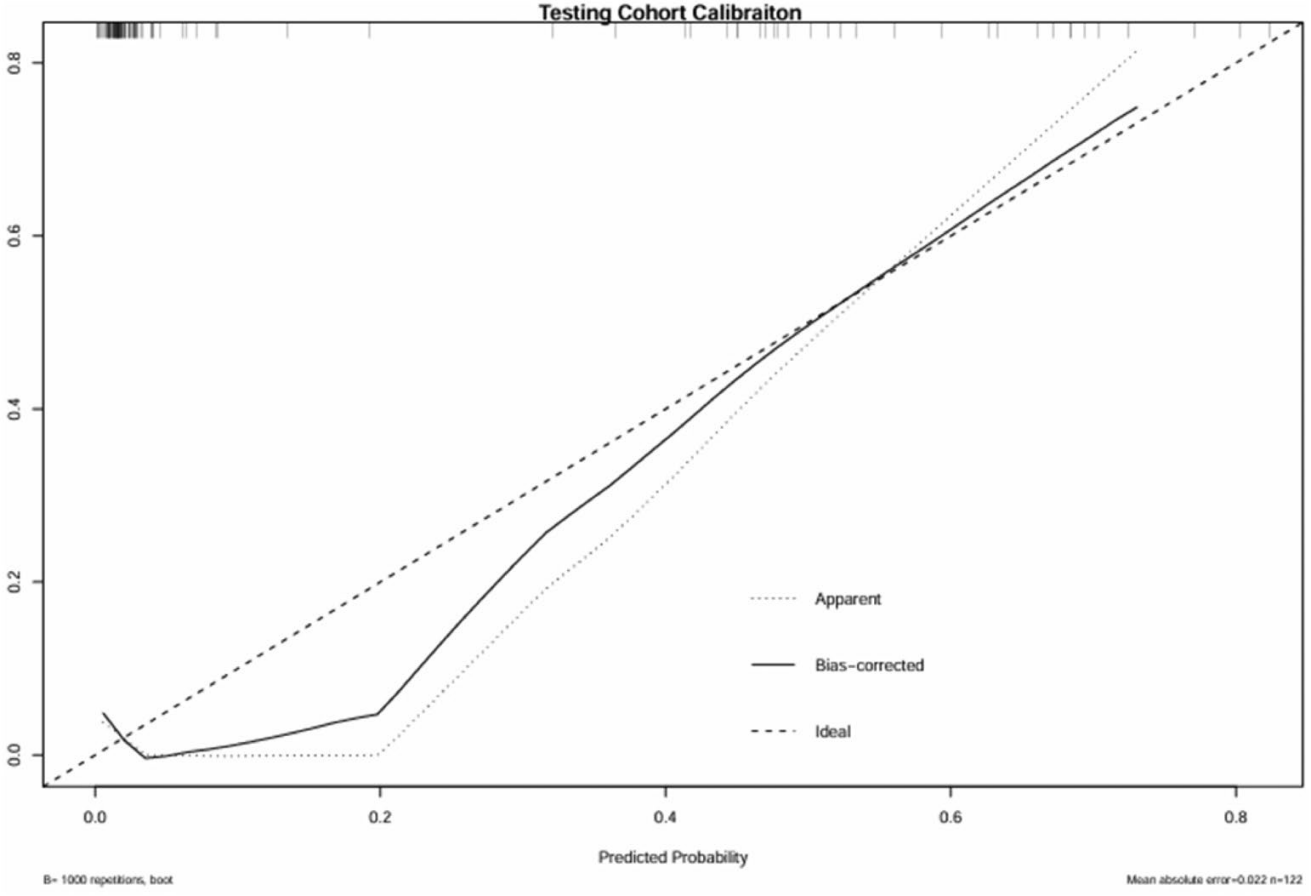
Calibration curve of the LightgBM model validation set.

**FIGURE 5:**
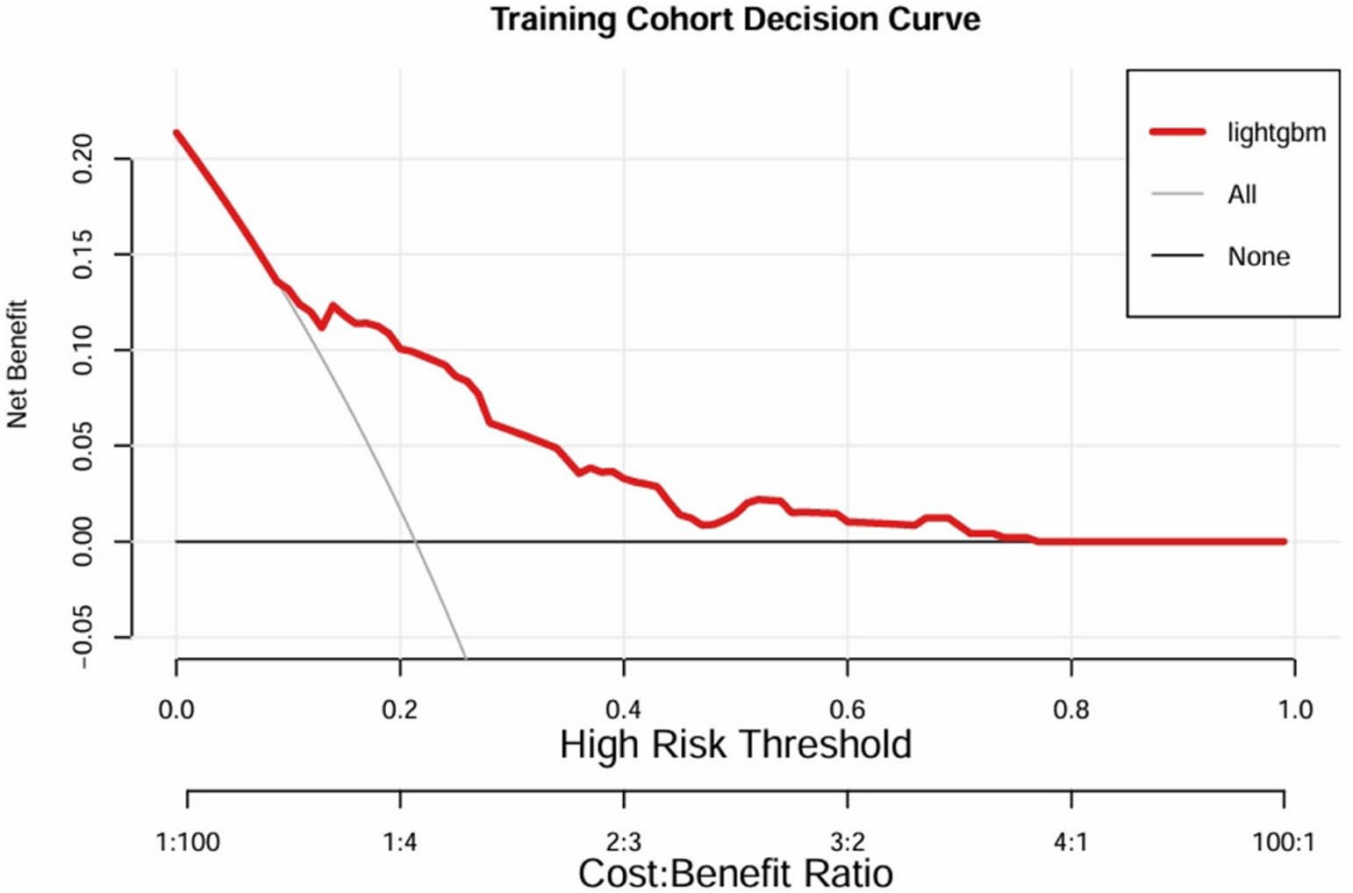
DCA of the LightgBM model training set.

**FIGURE 6:**
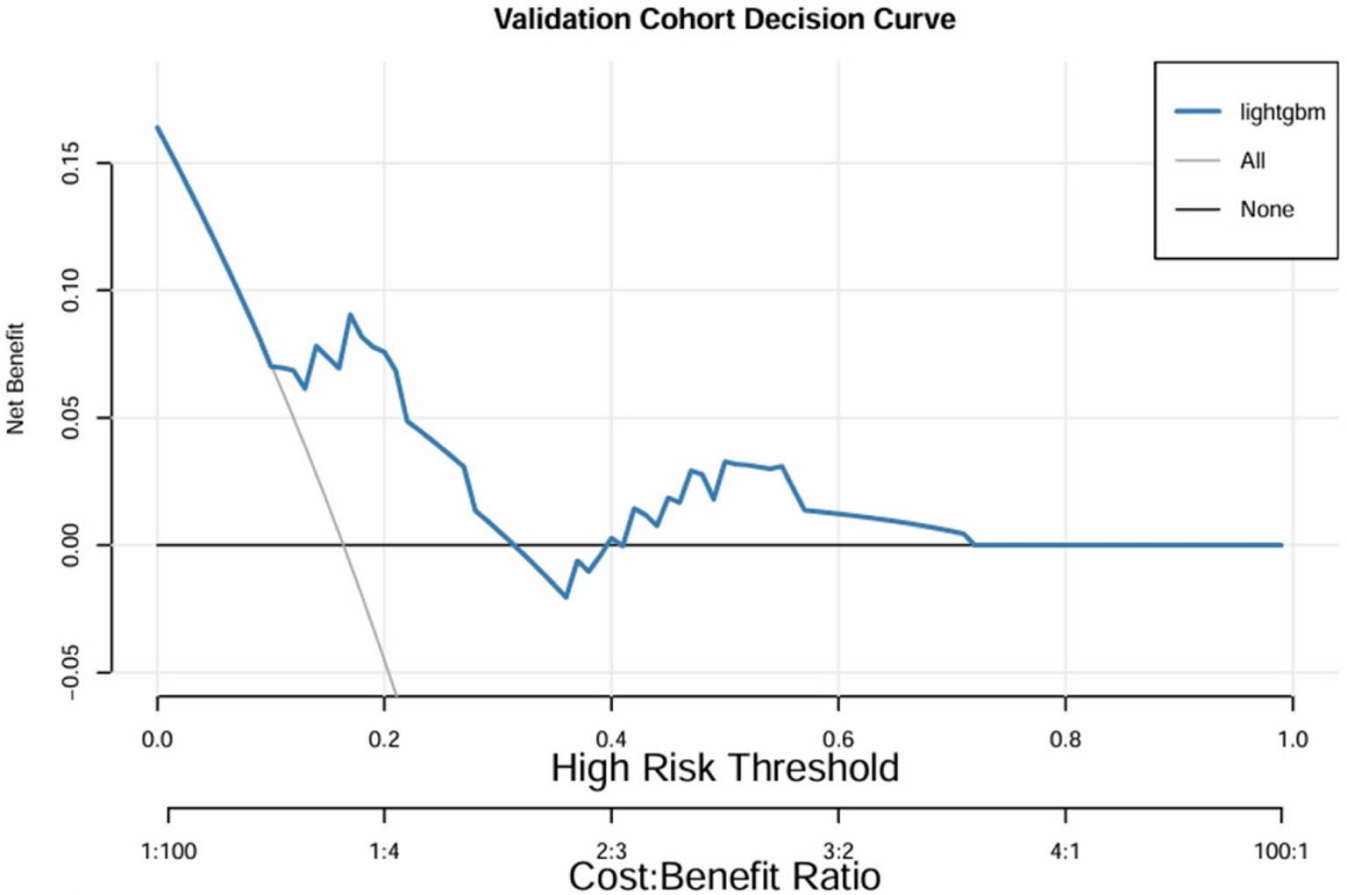
DCA of the LightgBM model validation set.

**FIGURE 7:**
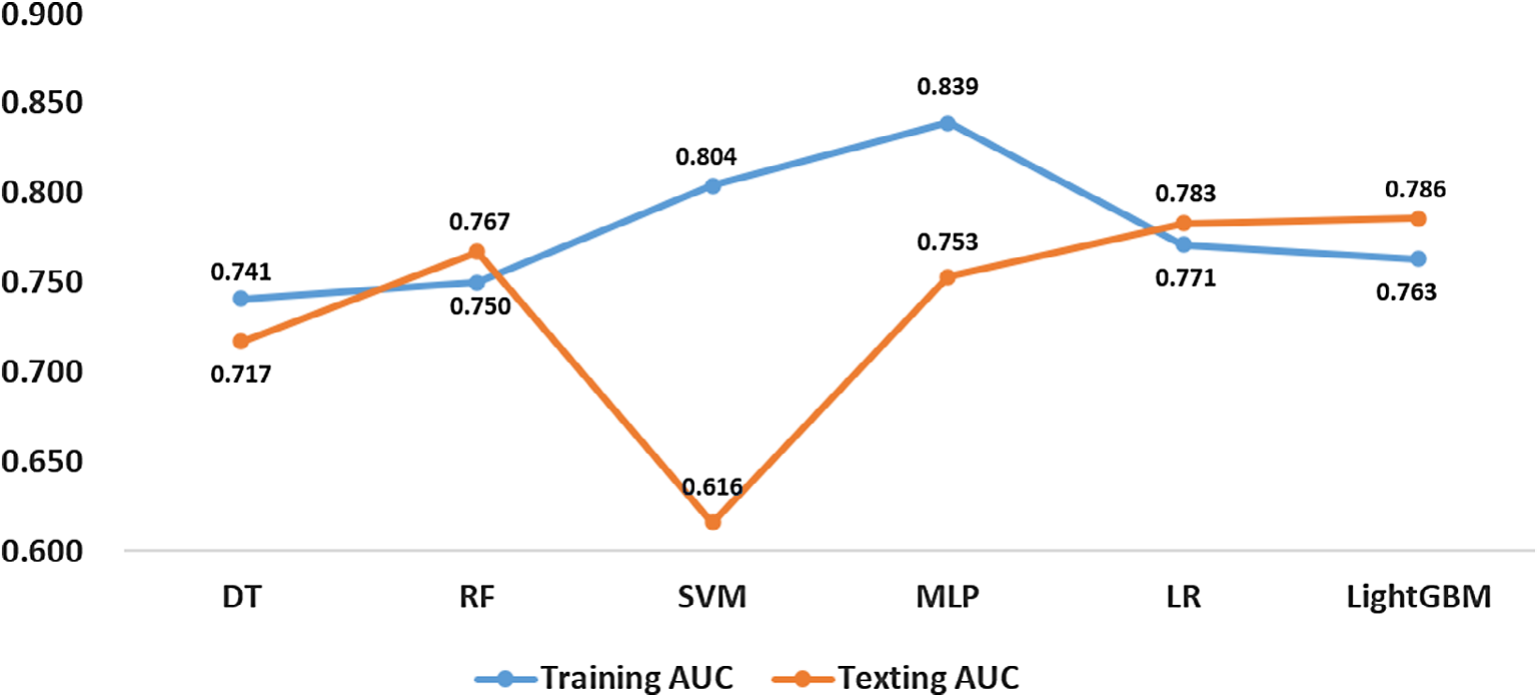
Comparison of AUC between the training set and the test set.

**FIGURE 8:**
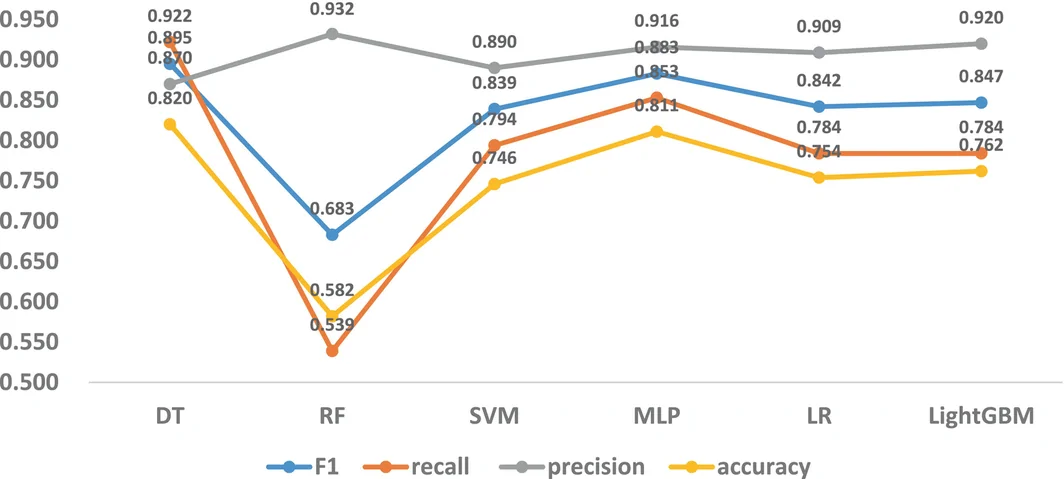
Comparison of Evaluation Metrics of test set models.

After the hyperparameter optimization and adjustment models were validated on the validation set, the predictive performance and overall performance of the six models were evaluated using the confusion matrix, AUC, accuracy, precision, recall, and F1 score. The confusion matrices and ROC curves for the models in the validation set are shown in Figures S1–S12. In the validation set, the AUC of the MLP and SVM algorithms was 0.753 and 0.616, respectively. Compared with other algorithms, their predictive performance when evaluated on unseen data was relatively average, and there was a significant gap between the AUCs of their training and test sets. It indicates that, after parameter tuning, the model still exhibits some overfitting and has relatively poor generalization ability. The AUC of the RF algorithm is 0.767, but the recall rate and accuracy are relatively poor. The AUC values of DT and LR were 0.717 and 0.783 respectively. After comprehensively evaluating the five model evaluation indicators, it was found that LightGBM performed best for predicting the risk of poor postoperative prognosis in patients with PDR. The AUC of this model on the validation set is 0.786, and all other indicators are at the average level, demonstrating good predictive performance and stability. The calibration curve (Figures 3 and 4) for the LightGBM model's training and validation set also indicated good calibration. The DCA (Figures 5 and 6) of the validation set showed that the LightGBM model yielded clinical net benefit at low‐to‐moderate risk thresholds, outperforming both the ‘treat‐all’ and ‘treat‐none’ strategies. However, compared with an ideal smooth DCA curve, the model's performance on the validation set exhibited some fluctuation, particularly with net benefit approaching zero in the moderate threshold range, suggesting limited discriminative ability for moderate‐risk patients, which may be attributable to sparse sample distribution in this interval or decreased model calibration. A detailed comparison of the evaluation metrics of six machine learning algorithms is shown in Figures 7 and 8.

### 3.6 Model Interpretation and Visualization

**FIGURE 9:**
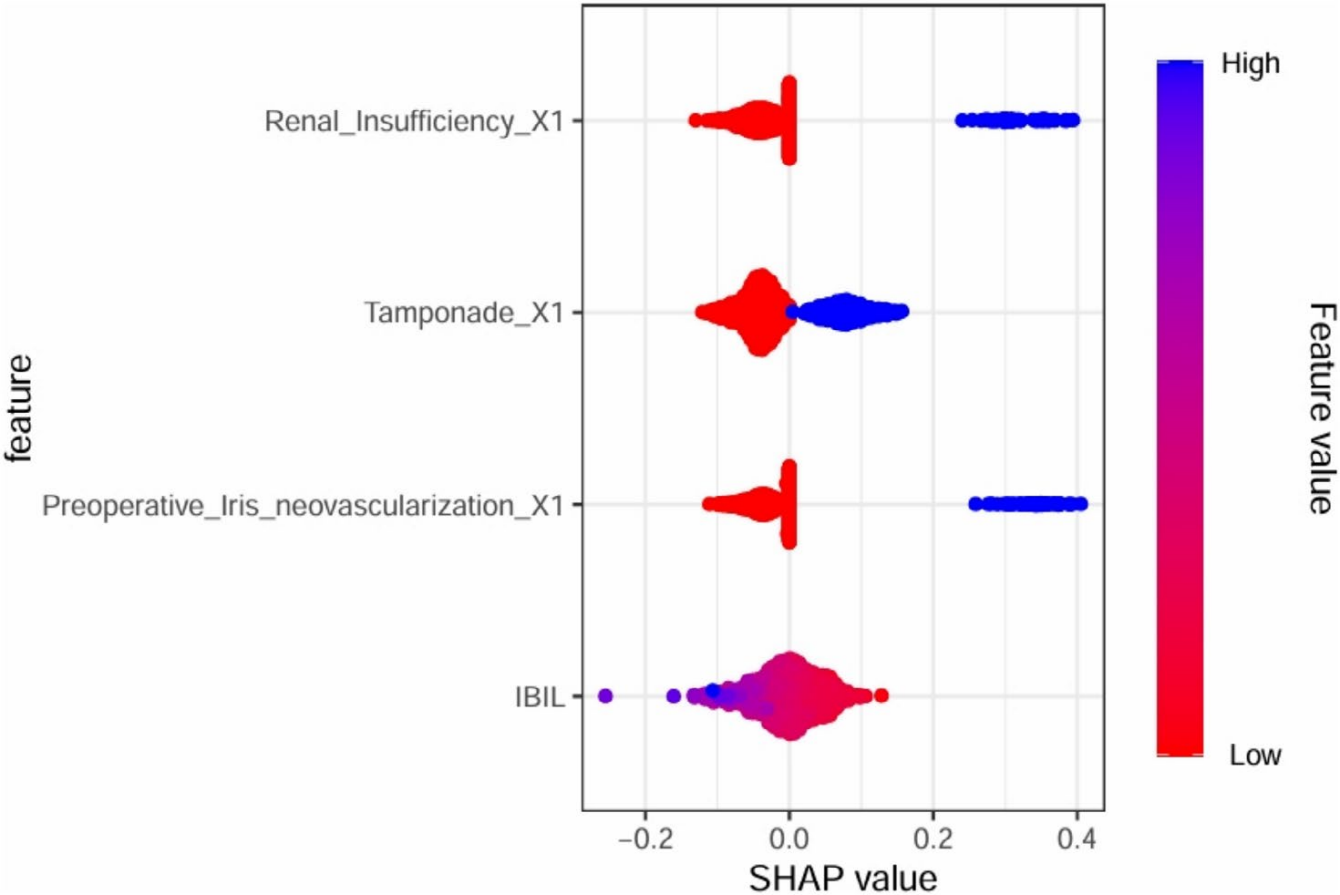
The SHAP diagrams of all variables.

As a model‐independent local interpretation framework, SHAP can quantify the contribution of each feature to the model's prediction results in the sample by calculating the SHAP value of the feature, and present the relationship between the feature and the prediction in a visual form, as shown in Figure 9. IBIL is a protective factor for poor postoperative prognosis. Renal Insufficiency, tamponade silicone oil, and preoperative iris neovascularization are risk factors for poor postoperative prognosis; that is, these characteristics will increase the predictive probability of poor prognosis. To make the LightGBM model more clinically applicable, a web calculator was initially developed in this study to visually improve its application, as detailed in Figure S13. After the feature values corresponding to the model are input and submitted, the web calculator can automatically display the results of the LightGBM model in predicting the risk of poor prognosis.

## 4 Discussion

The study shows that renal insufficiency, Preoperative iris neovascularization, preoperative Anti VEGF, tamponade silicone oil and IBIL are all factors associated with poor visual prognosis. Based on this, we constructed six machine learning models and ultimately determined that LightGBM was the optimal model. We also analysed the impact of predictive variables on patients with PDR after PPV.

As most patients who receive PPV are already in the severe stage of PDR, the postoperative prognosis shows complex and variable characteristics [16, 17]. Therefore, it is necessary to implement early intervention measures for patients at risk before surgery to delay the progression of the disease and help reduce blindness caused by complications of PDR. Based on this, it is particularly crucial to conduct early screening and accurately identify patients who have risk factors for a poor prognosis.

Guo et al. [18] screened predictive factors through multivariate logistic regression and constructed a predictive model for whether PDR patients would develop long‐term low vision after receiving PPV. However, in this study, only preoperative characteristics were included, and the sample size was relatively small, leading to insufficient reliability in the research results. Lee et al. [13] constructed a machine learning prediction model for poor visual outcomes after PPV in patients with PDR. The results show that the integrated decision tree and SVM algorithm perform well in this model. However, this study did not include factors such as diabetes duration and serum albumin concentration that might affect postoperative visual acuity outcomes, thereby limiting the model's predictive accuracy. In contrast, this study conducted a more comprehensive screening of predictive factors. Among them, the best‐performing model, LightGBM, demonstrates superior sensitivity, balance, and predictive performance compared to the aforementioned models.

Renal insufficiency is a risk factor for poor postoperative prognosis in patients with PDR, which is consistent with the research results of Li Xuejing et al. [19]. Impaired renal function is a sign of increased vulnerability to adverse health events, which helps identify patients with a greater risk of severe postoperative visual complications, thereby formulating more targeted and effective management strategies [20]. A lower eGFR can impair toxin clearance, leading to the accumulation of uremic compounds such as sulfate indole. These compounds exacerbate retinal hypoxia by amplifying inflammation and oxidative stress, while increasing VEGF levels. Elevated VEGF can drive abnormal growth of retinal blood vessels. Meanwhile, fluid retention due to renal insufficiency and hypertension can increase intraretinal pressure, aggravate vascular leakage, and ultimately promote further disease progression and affect the prognosis of patients [21]. Dynamic monitoring of renal function indicators and early intervention have crucial clinical significance for preventing adverse outcomes such as glomerular hyperfiltration [22]. Therefore, in addition to local ophthalmic treatment, patients with PDR also need to combine drug treatments such as blood sugar control, blood pressure management, and lipid regulation, as well as dietary control after surgery. At the same time, they should actively treat underlying diseases such as kidney disease. These comprehensive treatment measures are conducive to stabilizing the overall condition of patients and thereby improving their postoperative prognosis. Among them, the improvement of renal function is of great significance to the prognosis of patients with PDR.

We screened out the risk factors affecting prognosis, which were consistent with previous studies [23, 24, 25, 26, 27]. Preoperative presence of iris neovascularization and intraoperative silicone oil filling will increase the risk of poor prognosis for patients. Relevant studies have shown that among patients with PDR, approximately 65% will develop iris neovascularization, and among them, 20% will progress to neovascular glaucoma [28]. Patients with diabetic retinopathy have long‐term ischemia and hypoxia in the retina, which activates hypoxia‐inducible factors and stimulates excessive secretion of angiogenic factors (VEGF), leading to fragile and easily leaking and bleeding iris neovascularization [29, 30]. Neovascularization appears on the surface of the iris, combined with the contraction and traction of the fibrovascular tissue in the anterior chamber angle, leading to adhesion and closure of the iris and the anterior chamber angle, obstruction of aqueous humour circulation, and increased intraocular pressure. This results in neovascular glaucoma, causing severe eye pain or headache in patients, damage to the optic nerve, and a sharp decline in vision. In severe cases, it can even lead to blindness [31, 32, 33].

Patients who received intraoperative intraocular silicone oil (SO) tamponade exhibited a higher risk of poor postoperative visual prognosis. This observed association should be interpreted with caution, as it may reflect underlying indication confounding: SO is preferentially used for more severe cases, such as those presenting with tractional retinal detachment, which inherently carry a higher baseline risk of unfavourable visual outcomes. On one hand, SO may act as an independent biological driver of poor prognosis: it can dissolve lipophilic components in the retina and interfere with the photoprotective effect of lipophilic macular pigments on the macula [34]; on the other hand, emulsification and displacement of SO can exert chronic mechanical pressure on the retinal surface, inducing abnormal retinal function and subsequent vision loss [35]. These mechanisms may collectively contribute to macular dysfunction, potentially leading to suboptimal postoperative visual recovery. In addition, the prognostic significance of SO in the unadjusted analysis may also serve as a surrogate marker for greater baseline surgical complexity and more advanced disease severity, rather than a direct causal factor.

TBIL is the end product of systemic haemoglobin catabolism, consisting of IBIL and DBIL, and IBIL constitutes more than 95% of total bilirubin in healthy adults [36]. Studies have shown that low bilirubin levels are significantly associated with an increased risk of DR [37]. Existing studies predominantly focus on the association between TBIL and DR, while limited research explores the predictive efficacy of serum IBIL for DR progression and postoperative prognosis [38, 39]. Our study revealed that preoperative IBIL exhibited superior predictive value for postoperative prognosis in PDR patients. We further identified that IBIL concentration was negatively correlated with adverse postoperative outcomes, acting as an independent protective factor for PDR prognosis.

Previous studies have similarly demonstrated that IBIL serves as an intermediate product of haemoglobin metabolism and stands out as one of the most potent endogenous antioxidants. It possesses a potent antioxidant capacity, enabling it to neutralize free radicals and suppress oxidative reactions, thus providing protection to cells. A slight elevation in its levels can decrease intracellular oxidative stress, enhance the cellular metabolic environment, boost insulin sensitivity, and regulate blood glucose metabolism. Consequently, this can slow down disease progression, reduce the occurrence of complications, and improve the prognosis [40]. Notably, the protective prognostic role of IBIL observed in our study is biologically plausible yet requires further rigorous validation. First, future analyses should explore potential nonlinear relationships between IBIL levels and PDR postoperative outcomes to clarify the dose‐effect correlation. Second, it is necessary to verify whether the protective effect of IBIL is mediated by systemic anti‐inflammatory biomarkers, given the tight interplay between oxidative stress and inflammation in DR pathogenesis. Third, sensitivity analyses are warranted to exclude the confounding effect of extreme outliers and confirm the robustness of the association between IBIL and PDR prognosis. Therefore, incorporating preoperative serum indirect bilirubin detection into the routine monitoring index system for PDR patients helps medical staff have a deeper understanding of the patients' conditions before surgery, predict potential risks that may occur during the postoperative recovery process in advance, and formulate and take targeted intervention measures on time, thereby effectively improving the prognosis of patients.

In addition, machine learning algorithms exhibit a black‐box effect, making it difficult to quantify the impact of factors on disease, which poses a challenge for clinical application [41]. This study used it to construct a predictive model, quantifying and visualizing the LightGBM model with the best performance through SHAP graphs and web calculators, making it more in line with the practical needs of clinical practice and enabling rapid screening of prognostic risks after PPV in PDR patients. In medical care, it can help medical staff identify potential adverse events in advance by using data, implement personalized interventions, reduce the risk of poor outcomes, and improve patients' quality of life.

A limitation of this study is that the data originated from the same hospital's retrospective medical records. The risk prediction model constructed was only internally trained and validated, and no external validation was conducted. Subsequent research can conduct large‐sample, multi‐centre prospective studies to further evaluate the predictive performance and accuracy of the model, thereby enabling its application in real‐world clinical data.

## 5 Conclusion

In conclusion, the development of a machine learning‐based prognostic risk prediction model for long‐term visual outcomes following PPV in PDR patients may assist clinicians in identifying patients at higher risk of poor visual prognosis. This could potentially support more individualized follow‐up planning. Further prospective validation and cost‐effectiveness analyses are warranted to confirm its clinical utility and impact on healthcare resource allocation.

## Author Contributions

**Yan Gao:** resources, writing – review and editing. **Jinghua Shi:** funding acquisition, supervision, resources, writing – review and editing. **Tingting Jia:** funding acquisition, writing – review and editing. **Xiaolu Wang:** conceptualization, methodology, software, data curation, investigation, writing – original draft. **Tiantian Li:** data curation, investigation. **Xuehong Han:** data curation, investigation. **Lixia Guo:** writing – review and editing, funding acquisition. **Yan Wang:** funding acquisition, writing – review and editing.

## Funding

Research Fund Project of Shanxi Eye Hospital (C202403).

## Conflicts of Interest

The authors declare no conflicts of interest.

## Supporting information


**Table S1:** Variable encoding.
**Figure S1:** DT Model confusion matrix.
**Figure S2:** DT Model ROC curve.
**Figure S3:** RF Model confusion matrix.
**Figure S4:** RF Model ROC curve.
**Figure S5:** SVM Model confusion matrix.
**Figure S6:** SVM Model ROC curve.
**Figure S7:** MLP Model confusion matrix.
**Figure S8:** MLP Model ROC curve.
**Figure S9:** LR Model confusion matrix.
**Figure S10:** LR Model ROC curve.
**Figure S11:** LightGBM Model confusion matrix.
**Figure S12:** LightGBM Model ROC curve.
**Figure S13:** LightGBM Model Web calculator.

## Data Availability

The data that supports the findings of this study are available in the Supporting Information of this article.
