# Supplementary material for: Construction and Validation of a Long‐Term Visual Prognosis Prediction Model for Proliferative Diabetic Retinopathy After Vitrectomy Based on Machine Learning
Source: Endocrinol Diabetes Metab. 2026 Sep 7;9(5):e70314. doi: 10.1002/edm2.70314 (PMC13550875; doi:10.1002/edm2.70314)
Supplement: Supplementary file 1 [file EDM2-9-e70314-s001.docx]

**Construction and validation of a long-term visual prognosis prediction modelfor proliferative diabetic retinopathy after vitrectomy based on machine learning**

**Xiaolu Wang, Jinghua Shi, Yan Gao, Tiantian Li, Xuehong Han, Lixia Guo, Tingting Jia, Yan Wang**

**Content**

**1. Supplementary TableS1.Variable encoding.…………..………….…..…….…..2**

**2. Supplementary FigureS1-S12.Confusion matrices and ROC curves of the validation sets of six machine learning algorithm models.………………..…......5**

**3. Supplementary FigureS13.LightGBM Model Web calculator………….......10**

# 1.

**TableS1.Variable encoding.**

|  | **classification** | **variable** **name** | **types of variables** | **Encoding of categorical variables** | **Data Missing Rate（%）** |
| --- | --- | --- | --- | --- | --- |
| 1 | **general data** | Age（years） | continuous |  | 0.0 |
| 2 |  | Sex | dichotomy | 0: female 1: man | 0.0 |
| 3 |  | Marital_Status | dichotomy | 0: married  1: Unmarried/Divorced/widowed | 0.0 |
| 4 |  | Residence | Unordered multi-classification | 0: country  1:town  2: city | 0.0 |
| 5 |  | Career | Unordered multi-classification | 0: peasant  1: staff  2: freelance work  3: retirement  4: unemployed | 0.0 |
| 6 |  | Insurance_Type | Unordered multi-classification | 0: worker's insurance  1: resident insurance  2: New Rural Co-operative Medical System  3: self-paying | 0.0 |
| 7 |  | Main_Caregiver | Unordered multi-classification | 0: spouse  1: offspring  2: solitude  3: else | 0.0 |
| 8 |  | Systolic_Blood_Pressure（mmHg） | continuous |  | 0.2 |
| 9 |  | Diastolic_Blood_Pressure（mmHg） | continuous |  | 0.2 |
| 10 |  | Body_Mass_Index（kg/m2） | continuous |  | 19.2 |
| 11 |  | Diabetes_Type | dichotomy | 0:Ⅰtype  1:Ⅱtype | 0.0 |
| 12 |  | Diabetes_Duration（years） | continuous |  | 9.0 |
| 13 |  | Age_at_diabetes_diagnosis（years） | continuous |  | 13.8 |
| 14 |  | Diabetes_Treatment | Unordered multi-classification | 0: Take oral medication alone  1: Insulin alone  2: Oral medication combined with insulin | 7.1 |
| 15 |  | Hypertension | dichotomy | 0:No 1:Yes | 0.8 |
| 16 |  | CHD | dichotomy | 0:No 1:Yes | 0.8 |
| 17 |  | Cerebral_Infarction | dichotomy | 0:No 1:Yes | 0.8 |
| 18 |  | Renal_Insufficiency | dichotomy | 0:No 1:Yes | 0.8 |
| 19 | **Preoperative risk factors** | Eye | dichotomy | 0: left eye  1: right eye | 0.0 |
| 20 |  | Staging_Of_DR | Ordered multi-classification | 0:IV stages  1:V stages  2:VI stages | 0.0 |
| 21 |  | Preoperative_Contralateral_BCVA（LogMAR） | continuous |  | 0.0 |
| 22 |  | Preoperative_IOP(mmHg) | continuous |  | 0.0 |
| 23 |  | Preoperative_Iris_neovascularization | dichotomy | 0:No 1:Yes | 0.0 |
| 24 |  | Preoperative_Vitreous_Hemorrhage | dichotomy | 0:No 1:Yes | 0.0 |
| 25 |  | Preoperative_Tractional_Retinal_Detachment | dichotomy | 0:No 1:Yes | 0.0 |
| 26 |  | Preoperative_PRP | dichotomy | 0:No 1:Yes | 0.0 |
| 27 |  | Symptom_Duration(month) | continuous | 0:No 1:Yes | 7.1 |
| 28 | **Intraoperative risk factors** | Combined_Cataract_Extraction | dichotomy | 0:No 1:Yes | 0.0 |
| 29 |  | Intraoperative_PRP | dichotomy | 0:No 1:Yes | 0.0 |
| 30 |  | Intravitreal_Injection | dichotomy | 0:No 1:Yes | 0.0 |
| 31 |  | Tamponade | Unordered multi-classification | 0:No  1: Silicone oil  2: Gases | 0.2 |
| 32 | **biochemical criterion** | HbA1c(%) | continuous |  | 11.3 |
| 33 |  | Monocyte(x10^9/L) | continuous |  | 0.2 |
| 34 |  | Lymphocyte(x10^9/L) | continuous |  | 0.2 |
| 35 |  | Platelet(x10^9/L) | continuous |  | 0.2 |
| 36 |  | Hematokrit(g/L) | continuous |  | 0.2 |
| 37 |  | Hemoglobin(g/L) | continuous |  | 0.2 |
| 38 |  | Fasting_Blood_Glucose(mmol/L) | continuous |  | 0.2 |
| 39 |  | Uric_acid(ummol/L) | continuous |  | 1.1 |
| 40 |  | Creatinine(ummol/L) | continuous |  | 1.3 |
| 41 |  | eGFR(ml/min/1.73m^2^) | continuous |  | 1.3 |
| 42 |  | Urea | continuous |  | 1.3 |
| 43 |  | Serum_albumin(g/L) | continuous |  | 0.8 |
| 44 |  | Serum_total_protein(g/L) | continuous |  | 0.8 |
| 45 |  | Sap(U/L) | continuous |  | 0.8 |
| 46 |  | IBIL(µmol/L) | continuous |  | 0.9 |
| 47 |  | DBIL(µmol/L) | continuous |  | 0.8 |
| 48 |  | TBIL(µmol/L) | continuous |  | 0.8 |
| 49 |  | Serum_alanine_aminotransferase(U/L) | continuous |  | 3.4 |
| 50 |  | Serum_aspartate_aminotransferase(U/L) | continuous |  | 0.8 |
| 51 |  | Serum_b_glutamyl_transferase(U/L) | continuous |  | 0.8 |
| 52 |  | Prothrombin_activity | continuous |  | 0.7 |
| 53 | **outcome indicator** | Visual_prognosis | dichotomy | 0: good 1: poor |  |

# 2.Confusion matrices and ROC curves of the validation sets of six machine learning algorithm models

（1） Decision tree（DT）

After building the decision tree and making predictions, the confusion matrix is shown in FigureS1.The model's accuracy was 0.820, precision 0.870, recall 0.922, F1 score 0.895, and AUC 0.717 (95% CI: 0.587~0.842).The ROC curve appears in Figure S2. Based on these results, the decision tree model had good recall and F1 score.

|  |  |
| --- | --- |
| FigureS1 DT Model confusion matrix. | FigureS2 DT Model ROC curve. |

（2）Random Forest（RF）

The results showed that the random forest model's accuracy on thetest set was 0.582, precision was 0.932, recall was 0.539, and F1 score was 0.683. The AUC was 0.767(95%CI: 0.633~0.879). The random forest model shows poor accuracy and recall. The confusion matrix of the random forest is plotted in FigureS3, andthe ROC curve is shown in FigureS4.

|  |  |
| --- | --- |
| FigureS3 RF Model confusion matrix. | FigureS4 RF Model ROC curve. |

（3） Support Vector Machine（SVM）

The trained SVM model was tested on thetest set, yielding an accuracy of 0.746, precision of 0.890, recall of 0.794, F1 score of 0.839, and AUC of 0.616(95% CI: 0.457~0.772). The model demonstrated good precision and F1 score. The ROC curve is shown in FigureS5, and the confusion matrix in FigureS6.

|  |  |
| --- | --- |
| FigureS5 SVM Model confusion matrix. | FigureS6 SVM Model ROC curve. |

（4） Multilayer Perceptron（MLP）

Results on the test set showed a prediction accuracy of 0.811, precision of 0.916, recall of 0.853, F1 score of 0.883, and AUC of 0.753(95% CI: 0.619~0.878), indicating good recall and relatively high sensitivity. FigureS7 shows the confusion matrix,and FigureS8 presents the ROC curve.

|  |  |
| --- | --- |
| FigureS7 MLP Model confusion matrix. | FigureS8 MLP Model ROC curve. |

（5） Logistic Regression（LR）

After testing, the results showed a prediction accuracy of 0.754, precision of 0.909, recall of 0.784, F1 score of 0.842, and AUC of 0.783(95% CI: 0.660~0.887). The model demonstrates good recall, high precision, and highAUC, as well as strong generalization and reliability. The confusion matrix is shown in FigureS9, and the ROC curve in FigureS10.

|  |  |
| --- | --- |
| FigureS9 LR Model confusion matrix. | FigureS10 LR Model ROC curve. |

（6） Light Gradient Boosting Machine（LightGBM）

After testing the LightGBM model on the test set, it achievedan accuracy of 0.762, precision of 0.920, recall of 0.784, F1 score of 0.847, and AUC of 0.786(95% CI: 0.663~0.891). The model's recall is good, and its AUC is high. FigureS11 shows the ROC curve, and FigureS12 shows the confusion matrix.

|  |  |
| --- | --- |
| FigureS11 LightGBM Model confusion matrix. | FigureS12 LightGBM Model ROC curve. |

# 3. Visualization of the best model

FigureS13 LightGBM Model Web calculator.
